# Significantly more intense pain in methadone-maintained patients who are addicted to nicotine

**DOI:** 10.18632/oncotarget.19222

**Published:** 2017-07-13

**Authors:** Lian-Zhong Liu, Lin-Xiang Tan, Yan-Min Xu, Xue-Bing Liu, Wen-Cai Chen, Jun-Hong Zhu, Jin Lu, Bao-Liang Zhong

**Affiliations:** ^1^ Affiliated Wuhan Mental Health Center (The Ninth Clinical School), Tongji Medical College of Huazhong University of Science and Technology, Wuhan, Hubei Province, China; ^2^ Mental Health Education Center, Central South University, Changsha, Hunan Province, China; ^3^ Department of Psychiatry, The First Affiliated Hospital of Kunming Medical University, Kunming, Yunnan Province, China

**Keywords:** pain, smoking, nicotine dependence, heroin dependence, methadone

## Abstract

Pain and cigarette smoking are very common in heroin-dependent patients (HDPs) receiving methadone maintenance treatment (MMT) and both have substantial negative effects on HDPs' physical and mental health. Nevertheless, very few studies have assessed the relationship between the two in HDPs. This study examined the association between pain intensity and smoking in Chinese methadone-maintained HDPs. A total of 603 HDPs were consecutively recruited from three MMT clinics in Wuhan, China, and administered with a socio-demographic and drug use questionnaire, a smoking questionnaire concerning average number of cigarettes smoked daily and Heaviness of Smoking Index, and Zung's Self-rating Depression Scale. We used a five-point Verbal Rating Scale to rate the intensity of pain. To determine whether pain's associations with smoking and nicotine dependence were independent, an analysis of covariance was adopted to control for the potential confounding effects of socio-demographic variables, drug use characteristics, and depressive symptoms. Net of potential confounders, in methadone-maintained HDPs, pain intensity was significantly higher in smoker than non-smokers (F = 11.836, *P* = 0.002) but the pain intensity did not differ significantly across patients with various levels of cigarette consumption (F = 1.992, *P* = 0.137), while the pain intensity significantly differed across patients with different levels of nicotine dependence (F = 3.252, *P* = 0.013). Pain is significantly associated with smoking in HDPs receiving MMT, this phenomenon may be explained by the association between nicotine dependence and pain.

## INTRODUCTION

There is evidence that pain is very prevalent among heroin-dependent patients (HDPs), for example, 39.7% HDPs treated in compulsory drug rehabilitation centers have chronic severe pain (CSP) [1] and 14.1% HDPs treated in methadone maintenance treatment (MMT) clinics have clinically significant pain [2]. Pain not only causes physical suffering, but also results in sleep disturbance and poor physical and mental quality of life in HDPs [1–4]. Therefore, pain is of great clinical concern in addiction treatment practice. However, in China, our knowledge on pain in HDPs is limited and the identification and treatment of pain-related problems are often neglected in clinical practice [1]. To the best of our known, only one recent study has characterized HDPs with CSP, which found that patients who were female, had a long duration of heroin use, and were depressed were more likely to have CSP [1]. Because this study recruited a small sample from a compulsory drug rehabilitation center, the generalizability of its findings is limited.

Cigarette smoking is also very common among HDPs [5], for example, over 90% HDPs in Chinese MMT clinics are current smokers, and approximately two-thirds of these smokers are heavy smokers [6]. Nevertheless, it remains unclear that why the smoking rate is so high among HDPs. The “self-medication hypothesis” argues that patients intake nicotine by smoking cigarettes to relieve negative emotional symptoms of protracted withdrawal, since the nicotine could partly mimic the function of certain excitatory transmitters such as dopamine and 5-hydroxytryptamine [7]. Considering that nicotine can produce an analgesic effect by activating Ach receptors [8], we hypothesized an association between smoking and pain in HDPs. However, very few Chinese studies have focused on such association, including those enrolled subjects other than psychiatric patients [9]. To deepen our understanding on the two clinical phenomena, the current study explored the relationship between smoking and pain with a sample of HDPs from MMT clinics.

## RESULTS

Of the 603 completers, there were 552 current smokers, the prevalence of current smoking in HDPs was 91.5%. These smokers smoked 18.2 cigarettes per day (standard deviation [SD] = 9.0, range = 3–50). The numbers of light, moderate, and heavy smokers were 144 (26.1%), 114 (20.7%), and 294 (53.3%), respectively. The Heaviness of Smoking Index (HSI) score of current smokers was 2.3 (SD = 1.5, range = 0–6). The numbers of smokers who were categorized as having no, mild, and moderate-to-severe nicotine dependence were 62 (11.2%), 389 (70.5%), and 101 (18.3%), respectively.

The pain intensity score of the whole sample was 2.8 (SD = 1.1). A total of 549 (91.0%) patients reported pain, of whom 465 (84.7%) had moderate or more intense pain.

Smokers had significantly more intense pain than non-smokers (2.8 ± 1.1 vs. 2.3 ± 1.2, *t* = 3.267, *P* < 0.001), however, results from univariate analysis within the sample of smokers showed that pain scores did not differ significantly across levels of cigarette consumption (light: 2.6 ± 1.2, moderate: 2.7 ± 1.1, heavy: 2.8 ± 1.1, F = 1.992, *P* = 0.137). Comparisons of characteristics between smokers and non-smokers revealed significant differences in terms of marital status, status of employment, heroin use duration, MMT duration and Zung's Self-rating Depression Scale (SDS) score (Table 1). After controlling for the confounding effects of these factors with analysis of covariance (ANCOVA), pain intensity score remained statistically significantly higher in smokers than non-smokers (F = 11.836, *P* = 0.002).

**Table 1:** Comparison of characteristics between smokers and non-smokers in MMT clinics*

Characteristics	Smokers (*n* = 552)	Non-smokers (*n* = 51)	Statistics	*P*
Gender: male	378 (68.5%)	34 (66.7%)	χ2 = 0.071	0.792
Age (years)	38.4 (7.5)	38.8 (8.7)	*t* = 0.359	0.721
Education year	10.8 (9.5)	9.1 (8.1)	*t* = 1.237	0.217
Marital status: never-married/separated/divorced/widowed	228 (41.3%)	10 (24.4%)	χ2=5.544	0.033
Unemployment	273 (49.5%)	17 (33.3%)	χ2=4.862	0.027
Heroin use duration (years)	11.3 (8.6)	8.7 (4.9)	*t* = 2.126	0.034
MMT duration (months)	24.6 (10.9)	12.1 (5.9)	*t* = 8.077	< 0.001
SDS	39.6 (8.5)	33.5 (7.7)	*t* = 4.941	< 0.001

*Continuous and categorical variables were expressed as mean (SD) and frequency (percentage), respectively.

Results from univariate analysis within the smokers showed that pain intensity scores significantly differed across levels of nicotine dependence (no: 2.5 ± 1.2, mild: 2.7 ± 1.1, moderate-to-severe: 3.1 ± 1.0, F = 4.797, *P* = 0.009). Comparisons of characteristics between smokers with different levels of nicotine dependence revealed significant differences in terms of gender, age, education year, heroin use duration, MMT duration and SDS score (Table 2). After controlling for the confounding effects of these factors with ANCOVA, differences in pain intensity scores remained statistically significant between smokers with different levels of nicotine dependence (F = 3.252, *P* = 0.039). Further post-hoc comparisons with LSD approach demonstrated that, smokers with moderate-to-severe nicotine dependence scored significantly higher on pain intensity than those with mild nicotine dependence (*P* = 0.001), and smokers with mild nicotine dependence scored significantly higher than those without nicotine dependence (*P* = 0.019). In addition, pain scores of non-smokers were comparable to smokers without nicotine dependence (2.3 ± 1.2 vs. 2.5 ± 1.2, *t* = 0.678, *P* = 0.533), but significantly lower than those of smokers with mild and moderate-to-severe nicotine dependence (*P* = 0.043 and *P* < 0.001).

**Table 2:** Comparison of characteristics between patients with different levels of nicotine dependence in MMT clinics

Characteristics	No dependence (*n* = 62)	Mild dependence (*n* = 389)	Moderate-to-severe dependence (*n* = 101)	Statistics	*P*
Gender: male	56 (90.3)	269 (69.2)	53 (52.5)	χ2 = 25.937	< 0.001
Age (years)	40.5 (6.6)	37.6 (6.7)	38.5 (8.1)	F = 4.183	0.016
Education year	8.3 (3.1)	9.5 (2.4)	10.5 (2.0)	F = 15.415	< 0.001
Marital status: never-married/separated/divorced/widowed	23 (37.1)	163(41.9)	42 (41.6)	χ2 = 1.042	0.594
Unemployment	28 (45.2)	187 (48.1)	58 (57.4)	χ2 = 3.322	0.191
Heroin use duration (years)	10.7 (4.8)	10.2(4.2)	8.5 (3.8)	F = 7.846	< 0.001
MMT duration (months)	23.4 (10.7)	25.9 (10.9)	20.6 (9.5)	F = 10.455	< 0.001
SDS	37.5 (9.3)	42.3 (10.6)	48.2 (14.0)	F = 10.876	< 0.001

*Continuous and categorical variables were expressed as mean (SD) and frequency (percentage), respectively.

## DISCUSSION

Smoking is a major cause for a variety of chronic conditions and increased mortality [6], and pain is also associated with many negative health outcomes of HDPs [2, 4, 10]. Therefore, analyzing the relationship between pain and smoking behavior of HDPs would help improve the physical and mental health of HDPs and facilitate the development of healthcare services provided by Chinese MMT clinics such as smoking cessation interventions.

The present study found that over 90% of HDPs were smokers and suffering from mild or more intense pain, and the majority of the smokers were moderate-to-severe nicotine dependent (74%) while the majority of the HDPs with pain had moderate or more intense pain (85%). Further adjustment analysis found that pain intensity of smokers was significant higher than nonsmokers, and the pain intensity increased with levels of nicotine dependence, not levels of cigarette consumption. Consistent with earlier studies [1–4, 6, 11], our study reported high prevalence rates of smoking and pain in methadone-maintained HDPs, however, the significant relationship between pain and nicotine dependence has rarely been reported in the literature.

Neurobiological research has found that nicotine could bind the nicotine acetylcholine receptors in the central nervous system and activate dopamine neurons in ventral tegmental area, prompting the release of excitatory neurotransmitters such as dopamine in nucleus accumbens, which further result in a sense of “pleasure” and other reward feelings in smokers [12]. This mechanism may explain the high rate of smoking in HDPs in MMT clinics: patients need to intake nicotine via smoking to relieve prolonged withdrawal symptoms of heroin dependence such as depression and anxiety [13]. The high rate of pain in HDPs could be related to impairments in endogenous opioid analgesic systems induced by long-term heroin use, for example, long term intake of exogenous opioid peptides would result in a low level of endogenous opioid peptides and a decreased tolerance of pain [14].

Given the analgesic effect of nicotine [8], a lower intensity of pain in smokers relative to non-smokers should be expected, but our findings showed the opposite. A previous study also reported significantly more depressive symptoms in smoking HDPs than non-smoking HDPs [13]. This unexpected result may be explained by the short half-life period of nicotine (1–2 hours), indicating the “pleasure” effect of nicotine is transient. In this case, HDPs need to continuously smoke to maintain the analgesic effect of nicotine. However, it is impossible for smokers to always smoke, therefore the positive effect of nicotine can not be maintained. It seems that smokers are more likely to be cases with intense pain because these patients need nicotine to relieve their pain, even if their pain can only be transiently relived by smoking. As a result of this, higher pain intensity was observed among smokers. In addition, some studies suggest that smoking could lower the pain threshold and make the smokers more sensitive to pain [15]. This might also explain the relationship between pain and smoking in HDPs.

There are a variety of reasons for smoking, including needs for social interaction, habitual behavior, “copying the smokers to make oneself seem mature”, or nicotine dependence, thus the smoking behavior of HDPs is influenced by many external factors other than nicotine intake. By contrast, nicotine dependence may be a more stable and specific proxy measure of nicotine needs in HDPs, so we found pain intensity was significantly related to levels of nicotine dependence, not cigarette consumption.

This study has several limitations. First of all, the design of this study is a cross-sectional survey, we can not determine the causality between smoking and increased pain intensity. Prospective follow-up studies are warranted to examine the possible causal relationship. Second, due to limited research resources, we did not measure the blood-nicotine level to explore the physiology mechanisms underlying the link between smoking and pain. Third, some other factors (i.e., use of alcohol and other drugs) that may contribute to the pain of HDPs were not assessed in the study so it is uncertain whether or not these factors would influence the smoking-pain association found in our study. Finally, the finding that pain intensity did not increase with levels of cigarette consumption, might be a result of small sample sizes of subgroups according to levels of cigarette consumption.

Despite of limitations, this study demonstrated the significant relationship between the nicotine dependence and pain in HDPs of MMT clinics. Findings from the present study suggest that a successful smoking cessation intervention may need to include effective pain management, and vice versa.

## MATERIALS AND METHODS

### Subjects

This was a secondary data analysis using database of our previous project “Mental health promotion for patients of MMT clinics in Wuhan, China: a comprehensive study”, which was conducted between 2009 and 2010. Criteria for subjects inclusion were: 1) 20 years and older, 2) met DSM-IV criteria for a lifetime diagnosis of heroin dependence, 3) receiving MMT, 4) able to complete the self-administered questionnaire, and 5) agreed to participate in the study. A total of 652 eligible patients were consecutively recruited and 603 completed the survey. The average age of the 603 patients was 38.1 years (SD = 7.0), and 69.8% were men. Detailed socio-demographic and substance use characteristics have been described elsewhere [16].

The Ethics Committee of Wuhan Mental Health Center approved the study protocol and all participants provided written informed consent before the beginning of the survey.

### Procedures and instruments

This was a self-administered questionnaire survey. The questionnaire consisted of the following parts: 1) Items requesting socio-demographic variables (i.e., age and gender) and drug use characteristics (duration of heroin use and MMT duration); 2) Characteristics of smoking, including whether the patient was currently smoking, the mean number of cigarettes smoked per day, duration of smoking, and HSI [17]. Current smokers referred to those who were currently smoking at least one cigarette per day and had smoked for at least six months [18]. According to the number of cigarettes per day, levels of cigarette consumption were stratified as light (1–10), moderate (10–19), and heavy (> 19) [18]. HSI is a commonly used measure of nicotine dependence, consisting of two items from the Fagerstrom Test for Nicotine Dependence: reported number of cigarette per day and time to first cigarette upon waking [17]. Each item was rated on a 4-point scale (0–3). The total score ranges from 0 to 6, with higher scores representing more severe nicotine dependence. Nicotine dependence is divided into three levels: no dependence (0), mild dependence (1–3), and moderate-to-severe dependence (4–6) [19]. The Chinese HIS is reliable and valid for Chinese smokers [19]. 3) Pain intensity was measured with the Five-point Verbal Rating Scale (VRS), asking “Overall, how intense is your pain now?”. The question has five-category responses: 1 = None, 2 = Mild, 3 = Moderate, 4 = Severe, 5 = Very severe. This measure of pain has been widely used in both clinical and population surveys and has been proved to be as valid as other common measures of pain intensity [20, 21]. 4) SDS [22]. This 20-item self-report scale was used to assess the severity of depressive symptoms using a four-point rating scale (1 = a little of the time to 4 = most of the time). Total SDS score ranges from 20 to 80, with higher scores indicating more severe depression. The Chinese SDS is reliable and valid for Chinese adult population [22, 23].

### Statistical analysis

Smoking characteristics and pain intensity of study subjects were described. Independent-samples *t*-test was used to compare pain scores between smoking and non-smoking subjects. Univariate analysis was used to compare pain scores between subjects with various levels of cigarette consumption and nicotine dependence. To determine whether the smoking-pain link was independent, an ANCOVA model that included variables that were unmatched between smokers and non-smokers as covariates was performed to control for the confounding effects of these unmatched variables [24, 25]. Similarly, an ANCOVA model that included factors that differed significantly across levels of nicotine dependence was also conducted to test pain's association with nicotine dependence. The least significant difference (LSD) method was used to test the pain intensity differences between groups with different levels of nicotine dependence. The statistical significance level was set at *p* < 0.05 (two-sided). SPSS software version 17.0 package was used for analyses.
